# Blink associated resetting eye movements (BARMs) are functionally complementary to microsaccades in correcting for fixation errors

**DOI:** 10.1038/s41598-017-17229-w

**Published:** 2017-12-04

**Authors:** Mohammad Farhan Khazali, Joern K. Pomper, Peter Thier

**Affiliations:** Department of Cognitive Neurology, Hertie Institute for Clinical Brain Research, University of Tübingen, Tuebingen, Germany

## Abstract

Blinks do not only protect the eye, but they do also correct for torsional eye position deviations by blink-associated resetting eye movements (BARMs). Although BARMs are functionally distinct from other eye movements in the torsional dimension, it has remained open if BARMs observed in the horizontal and vertical dimensions (fixational BARMs) are not simply microsaccades coinciding with blinks. We show here that fixational BARMs are functionally distinct and complementary to microsaccades in the following way: First, they compensate for large fixational error more efficiently than microsaccades, secondly, their probability to be executed in eccentric eye positions is higher, and thirdly, they reset the eyes into a position zone that is broader as compared to microsaccades. This suggests that BARMs help to keep the eyes in a working range wherein microsaccades guarantee high acuity vision. Moreover, we establish that fixational BARMs operate in a retina-centric frame.

## Introduction

Blinks do not only lubricate and protect the eye, but they do also correct for eye position deviations by blink-associated resetting eye movements (BARMs) as demonstrated in humans^1^. To bond blinks and the resetting eye movements in a synergy has the ecological advantage to keep the overall downtime of vision minimal as both, blinks and eye movements will compromise clear vision^2–5^. BARMs are functionally different from any other eye movement in the torsional dimension, in which they were primarily studied. However, it has remained open if BARMs observed in the horizontal and vertical dimension during fixation (fixational BARMs) are not in fact simply microsaccades coinciding with blinks. As yet, BARMs have only been studied in humans. Unfortunately, the limited amount of high resolution eye position data winnable with contact ring based search coils in humans, tolerated for short periods only, did not allow us to establish the existence of true fixational BARMs different from microsaccades beyond doubt. Moreover, this limitation also prevented the identification of the reference frame of operation. Here we report results made possible by eye movements recorded in non-human primates with permanently implanted, practically non-irritating 2D search coils, allowing the collection of the large amounts of data on fixational BARMs, needed to address the aforementioned questions.

Using this approach, we first of all demonstrate that BARMs are indeed functionally distinct eye movements also in the horizontal and vertical dimensions, complementing microsaccades in their effort to keep the eyes on target. Secondly, we establish that horizontal and vertical BARMs are organized in a retina-centered frame of reference with the fovea as their attractor, independent of the orientation of the eyes.

## Results

### Blinks reset the eye position also in non-human primates

**Figure 1:**
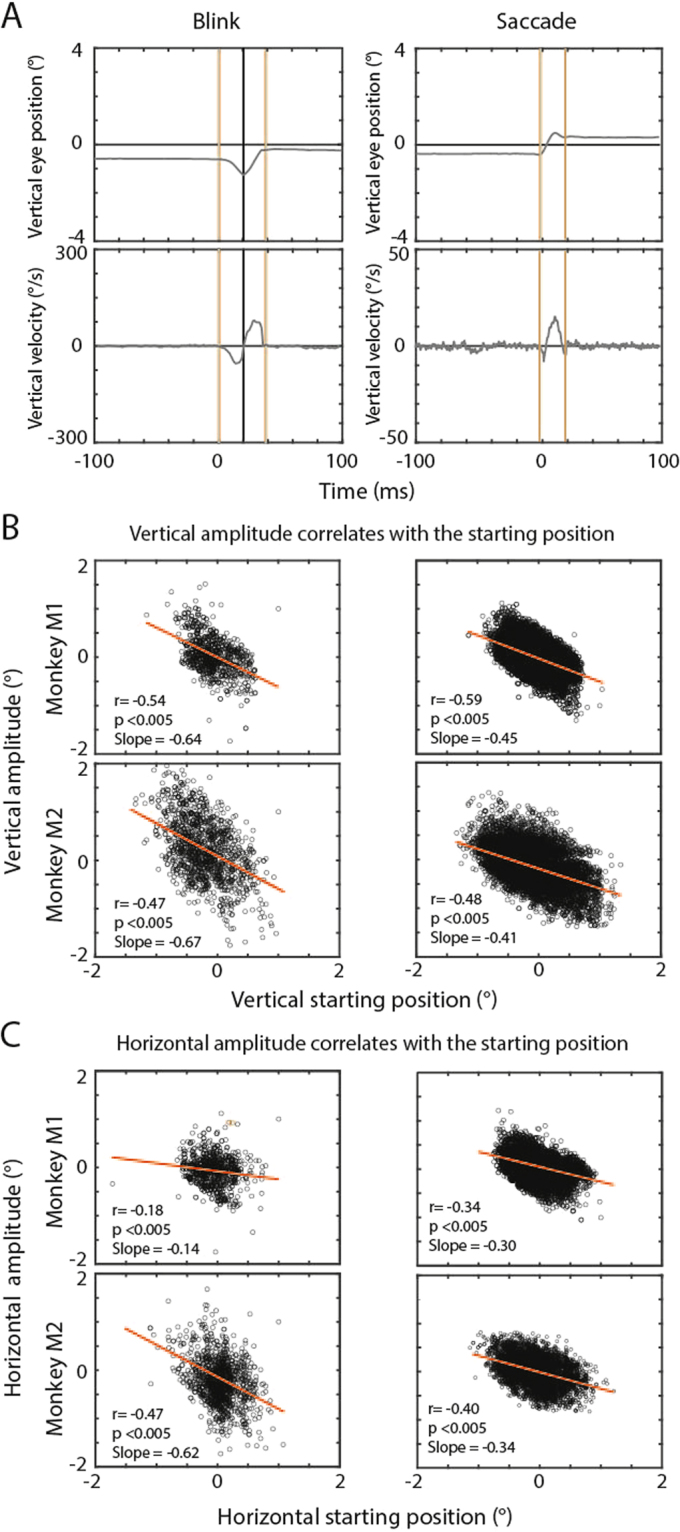
BARMs and microsaccades reset the eye position. (**A)** Left panel: exemplary eye position and velocity records documenting a blink occurring at t = 0. Note the typical negative-positive velocity alternation of a blink. The first component is given by a negative deflection of the velocity profile, the second one by a positive deflection. Right panel: a typical microsaccade is shown for comparison. Note that the velocity profile of a microsaccade has only one main peak, which allows its clear differentiation from blinks. (**B**) and (**C**) Plots of eye position shifts as a function of eye movement starting positions presented for each monkey separately (left column: blinks, right column: microsaccades). The vertical dimension is shown in (**B**) and the horizontal dimension in (**C**). The lines indicate the best fits yielded by linear regression analysis. All correlations are highly significant.

We trained two rhesus monkeys (M1 and M2) to fixate a white fixation dot (0.03° radius) on a black screen located straight ahead at a distance of 107 cm from the monkey (see Methods for more details). We identified blinks in the search coil records by a characteristic 2-component-sequence of eye position changes that have been described as blink-associated eye movements before and that are very different from the 1-component characteristic of saccades^6^. While fixating straight ahead a blink is associated with the eyes moving primarily downwards and towards the nose (first component) followed by a return towards the starting position (second component). This alternation of components is completed within 50 ms (Fig. 1A). The mean of the absolute amplitudes of the first component in the vertical dimension was 0.71° ± 0.72 (mean ± std), and 0.74° ± 0.76 for the second component for all blinks pooled from both monkeys. For the horizontal dimension the absolute amplitude of the first component was 0.65° ± 0.76 and for the second component was 0.53° ± 0.73. We found no significant difference between the absolute amplitudes of the first and the second components in neither horizontal nor vertical dimensions. The movement profiles of monkeys’ blink associated 2D eye movements were very similar to those of human fixational BARMs^1^. To identify these blink-associated eye movements also in non-human primates as fixational BARMs a resetting effect has to be shown. Hence, in analogy to our previous study in humans, we calculated a correlation between the blink starting eye position and the amount of eye position shift associated with a blink separately for the horizontal and the vertical dimension. As a matter of fact, we found the same kind of correlation in both monkeys (Fig. 1B,C) as in humans before. We conclude that also in non-human primates blink associated eye movements serve a resetting function dependent on the blink starting eye position; hence they qualify as fixational BARMs.

### BARM resetting differs from microsaccadic resetting

Microsaccades are small amplitude, rapid eye movements that shift the target image back into the center of the fovea in case of a preceding retinal error between the retinal target location and the center of the fovea^7^. The fixational BARMs observed in our monkeys have similar amplitudes, and they also compensate for this retinal error. Hence, could these BARMs actually be microsaccades that are yoked with blinks? The following findings imply a distinct identity of fixational BARMs and microsaccades (here operationally defined as saccades with amplitudes below 1.5°). First, we observed that the dependence of the amplitude of the fixational BARMs on the starting position differs from the corresponding dependence of the amplitude of microsaccades. The dependence of BARMs has a significantly (p < 0.005) steeper slope of 0.56° ± 0.015 (mean ± std) for vertical and 0.35° ± 0.027 for horizontal starting eye position as compared to 0.42° ± 0.096 and 0.17° ± 0.012 for the dependence of microsaccades (Fig. 1B,C). This difference in slopes means that blinks are on average more efficient in resetting than microsaccades. Yet, in view of the variance unexplained by the linear regression (Fig. 1B,C), we asked if the fixation dot is indeed better reached by BARMs as compared to microsaccades in the whole range of starting eye positions. Actually, on closer examination we found a higher efficiency of BARMs in contrast to microsaccades only for an outer zone of starting positions, beyond an eccentricity of 0.6°. Here the distance between the landing point and the fixation dot was smaller for BARMs (horizontal −0.0143° ± 0.023, vertical −0.33° ± 0.019 mean ± standard error of the mean) as compared to microsaccades (−0.1° ± 1.6e-04, −0.41° ± 1.9e-04, p < 0.005). This inferiority of microsaccades for an outer zone of starting positions was complemented by a superiority of microsaccades for an inner zone. When restricting the analysis to starting positions below 0.3° eccentricity, we observed a smaller standard deviation of landing points in microsaccades as compared to BARMs (horizontal 0.19° ± 0.014 versus 0.32° ± 0.005, vertical 0.28° ± 0.018 versus 0.38° ± 0.006, mean of standard deviations ± std of standard deviations, p < 0.005). In other words, fixational BARMs are able to efficiently move the target image into a comparatively broad zone around the fovea. Yet, within this zone they lack the precision of microsaccades.

**Figure 2:**
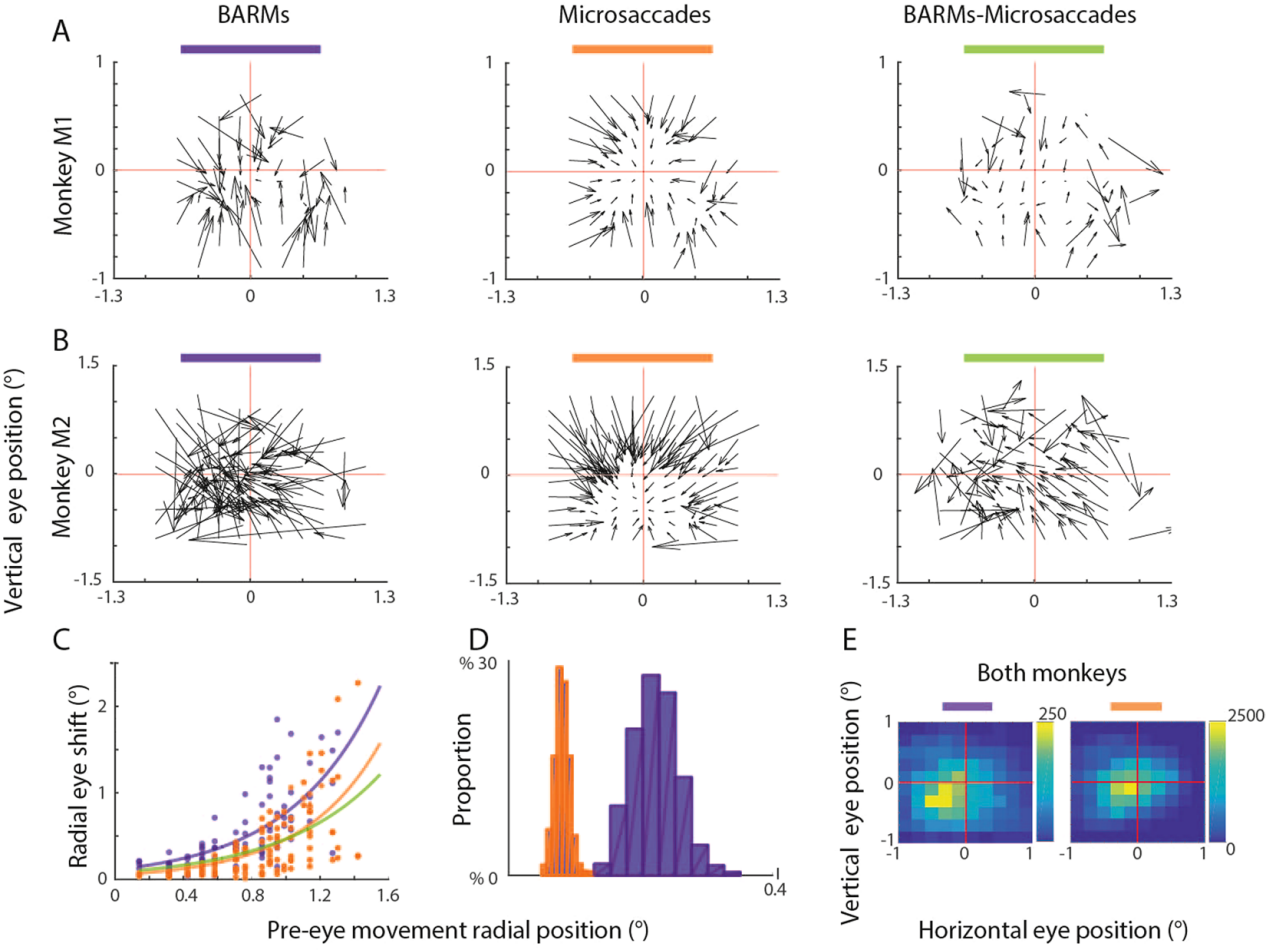
(**A**,**B**) Vector fields capturing the dependence of eye movement amplitude on starting position for the two monkeys. Each vector represent the mean movement amplitude based on all starting positions within spatial elements of 0.2° × 0.2°. The left panels depict the vector fields for BARMs, the middle ones those for microsaccades and the ones on the right show the subtraction fields (BARMs-microsaccades). (**C**) Depicts the dependence of vector length on the starting position. (**D**) Distribution of starting positions of BARMs and microsaccades compared. Blinks started at significantly (t-test, p < 0.005) larger eccentricity (0.22 ± 0.015) than microsaccades (0.08° ± 0.01°). Color conventions in (**C**,**D**)as defined in **(A**,**B**). (**E**) Spatial distribution of landing points of BARMs and microsaccades compared (spatial resolution 0.2 × 0.2°). BARMs landed at horizontal −0.15° ± 0.11°, vertical −0.25° ± 0.12° and microsaccades at horizontal −0.038° ± 0.083°, vertical −0.06 ± 0.096°; t-test comparison, p < 0.005.

This functional distinction of fixational BARMs and microsaccades is supported by the analysis illustrated in (Fig. 2). It compares the vector fields for fixational BARMs and microsaccades. Each vector represents the mean amplitude and direction of BARMs and microsaccades, respectively that were initiated from a square range of starting points (0.2° × 0.2°), in each case obtained by connecting the starting point and the endpoint of the movement. If fixational BARMs and microsaccades were identical, then subtracting the two vector fields should result in a zero pattern, perhaps overlaid by noise. This is clearly not the case. As shown in the right panels of (Fig. 2A,B), the subtraction field is non-zero and clearly converging. The fact that both BARMs and microsaccades are in principle fovea-oriented is also indicated by the clear dependence of the vector amplitudes (length) on the eccentricity of the starting position (Fig. 2C). However, typically BARMs originate from larger eccentricities (Fig. 2D) and cause larger changes in eye position towards the fovea than microsaccades (Fig. 2C).

Not only the dependency on eccentricity differs between fixational BARMs and microsaccades. Although both are centering eye movements, closer scrutiny reveals that also the distributions of landing points are slightly different with the one for fixational BARMs, see (Fig. 2E).

### Fixational BARMs are organized in retina-centered coordinates

**Figure 3:**
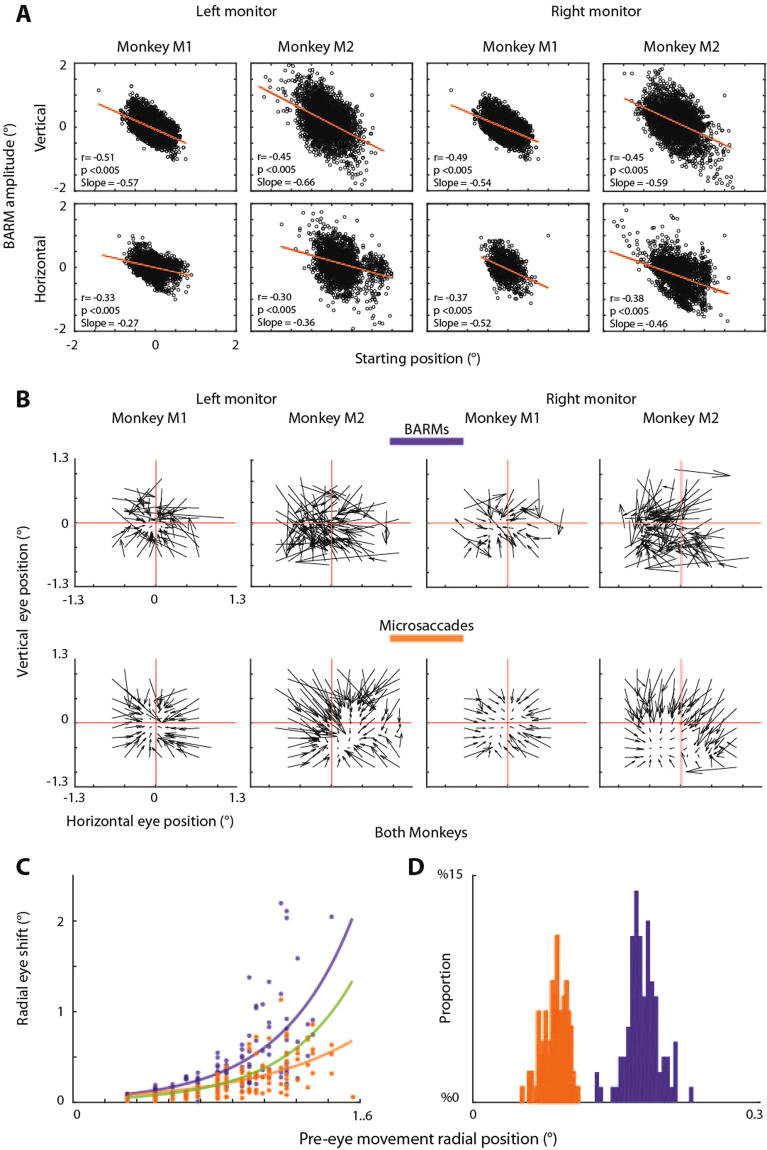
(**A**) Plots of BARMs induced eye position shift as function of the BARM starting position during eccentric fixation. Each dot represents one BARM in the vertical (first row) and in the horizontal dimension (second row). The two left columns represent fixation on the upper left monitor and the two on the right represent fixation on the upper right monitor. The lines indicate the best linear fits as derived from a regression analysis. All correlations are highly significant. (**B**) Resetting pattern of BARMs and microsaccades during eccentric fixation analogous to the plots in Fig. 2A and B. (**C**) Depicts the dependence of vector length on the starting position, the data are pooled from eccentric monitors and both monkeys. (**D**) Distribution of starting positions of BARMs and microsaccades compared; blinks started at significantly (t-test, p < 0.005) larger eccentricity (0.177 ± 0.018) than microsaccades (0.088° ± 0.012°). Color conventions in (**C**,**D**) as defined in (**A**,**B**).

The analysis discussed so far indicates that fixational BARMs try to move the retinal image of the target towards the fovea. In other words, these BARMs correct for a retinal error with the central retina as the point of reference. If the frame of reference of fixational BARMs were indeed retina centered, then moving the fixation dot away from straight ahead should still lead to fixational BARMs directed towards the fixation dot. We tested this prediction by comparing fixational BARMs collected when the fixation dot was straight ahead or, alternatively, located at 20° upward, 20° to the right or left, corresponding to an eccentricity of 28° each. We found in both monkeys that BARMs were directed towards the fixation dot independent of its position. In both eccentric positions the characteristics of resetting were the same as described for the fixation dot straight ahead before. There was a significant correlation between the size of the eye position shift and the blink starting eye position (Fig. 3A). The following features that justify a functional distinction between fixational BARMs and microsaccades during straight ahead fixation we also observed during eccentric fixation are: 1. The vector fields of BARMs and microsaccades illustrate a distinct distribution of resetting vectors (Fig. 3B), 2. The dependence of vector length on the starting position shows larger amplitudes for BARMs as compared to microsaccades (Fig. 3C), and 3. The distribution of starting positions confirms that BARMs are initiated at more eccentric eye positions with respect to the fixation dot as compared to microsaccades (Fig. 3D). In addition, also the distributions of landing points were slightly more eccentric for BARMs (mean of landing points: horizontal −0.13° ± 0.33, vertical−0.16° ± 0.13) than for microsaccades (mean of landing points horizontal −0.091° ± 0.13, vertical −0.1° ± 0.14; t-test comparison p < 0.005).

## Discussion

We have shown here that also in non-human primates blinks are associated with small resetting eye movements during fixation of a visual target that help to keep the target image on the fovea. These fixational BARMs are dependent on the pre-blink eye position, a feature they share with torsional BARMs^1^. Manipulating the position of the fixation dot clearly indicated that fixational BARMs operate in a retina-centered frame of reference. Whether the same frame of reference is also used in the control of torsional BARMs remains to be clarified. Fixational BARMs are functionally distinct from microsaccades. While fixational BARMs reset the eyes from a wider range of eccentricities into a quite broad zone close to the fovea, microsaccades are more precise within this zone, which suggests a complementary role of both. BARMs help to keep the eyes in a working range for microsaccades, thus facilitating the service of microsaccades to high acuity vision. This functionally distinct role of BARMs was primarily inferred from the analysis of data obtained during fixation of a straight ahead target and it is confirmed by the analysis of data obtained during fixation of eccentric targets.

The existence of eye movements during blinks that serve the purpose to reset the eyes in a retino-centred frame of reference suggests that patients suffering from a loss of saccades, might use blinks in order to acquire visual targets^8^. Whereas the case studies on these patients assumed that blinks would unveil saccades, otherwise lost, we would suggest against the backdrop of our study that the eye movements observed were actually fixational BARMS, arguably available as the underlying disease had spared their neuronal underpinnings, at least partially distinct from the one for saccades. Actually, the notion of a certain degree of specificity of the neuronal circuits of BARMs and saccades is also supported by the case of a patient reported by Hain and colleagues^9^ who generated hypermetric saccades, only when they blinked, immediately followed by a saccade in the opposite direction without intersaccadic interval. However valuable these case studies may be they cannot unravel the details of the microcircuitry underlying BARMs and how it differs from the one for (-micro) saccades. There is, for instance, the possibility that the neural motor circuitry involved in BARMs is largely congruent with the one for saccades. The functional differences may only arise through integration of separate signals (e.g. proprioceptive for BARMs and visual for saccade) to shape a spatial map for each of them. This would be compatible with the finding of differences in the neural processing between saccades and blinks in the superior colliculus^10^. To put it concisely, experiments at the level of single neurons have to follow to further explore the structural segregation between BARMs and microsaccades. The demonstration of fixational BARMs not only in humans but also in non-human primates is the prerequisite to set up such experiments.

## Methods

### Subjects

Two adult male rhesus monkeys (Macaca mulatta) took part in this study (M1: 10, M2: 8 years old). The experiments were approved by the local animal care committee (Protocol number: Regierungpräsidium Tübingen N6/13), conducted in accordance with German law and the Guidelines of the National Institutes of Health for the Care and Use of Laboratory Animals and carefully monitored by the veterinary administration (Regierungspräsidium and Landratsamt Tübingen). A magnetic scleral search coil was implanted into the right eye to record 2D eye position^11^ and a titanium head post to painlessly immobilize the head during experiments. For the sake of completeness, we mention that we also implanted a circular titanium chamber over occipital cortex for electrophysiological recordings not related to this study. All surgical procedures were conducted using aseptic techniques under full anesthesia consisting of isofluorane supplemented with remifentanil (1–2 μg/kg/min). All relevant physiological parameters such as body temperature, heart rate, blood pressure, pO_2_, and pCO_2_ were monitored. Postoperatively, buprenorphine was given until no sign of pain was evident. Animals were allowed to fully recover before starting the experiments.

### Experimental setup and behavior task

Three monitors (20 deg × 40 deg) were aligned at the same distance (107 cm) from the monkeys’ head (the central point between both eyes). The central monitor was at (0°, 0°) with respect to the straight ahead line of a monkey. The upper left monitor was centered at 20° left, 20° upward relative to straight ahead and the right monitor at 20° right and 20° upward. All monitors were oriented tangentially, i.e. their surface was perpendicular to the monkey’s line of sight. The optimal alignment was achieved by placing a laser light into the monitor’s center, perpendicular to its surface, that had to target a position exactly midway between the eyes. The monkeys were trained to maintain fixation of a white dot (0.03° diameter) on one out of the three black screens in a completely dark room. We measured the horizontal and vertical eye position using a home-made search eye coil system^12^ supporting a sampling rate of 1000 samples/s. The search coil signal was calibrated in 2D, separately for each monitor, by requiring the monkeys to maintain fixation of target dots presented at random in nine locations on a 3° × 3° grid with horizontal and vertical edges of 8° and a distance between neighboring horizontal or vertical positions of 2.66°. Each target dot, visible for 2 sec, had to be fixated at least 3 times. The monkeys had to maintain fixation within a 1° fixation window for 3 seconds continuously to get rewarded by a drop of juice or water. After the calibration, monkeys had to fixate the target on the central monitor for at least 10 minutes, and thereafter, they were required to fixate targets on the eccentric monitors, one after the other in random order across days.

### Fixation

The amount of variability of the fixation position, i.e. fixation noise, most probably the resultant of subtle fixation instability and electronic noise was very low (mean standard deviation ± std of 0.0058° ± 0.0021° for M1 and 0.0063° ± 0.0019° for M2). Both monkeys fixated the central target dot with a slight, yet significant mean downward and leftward eye position deviation from zero (nonparametric sign rank test, p < 0.05) (M1, vertical −0.1° ± 0.15°, mean ± std across sessions, horizontal −0.12° ± 0.16°), M2 fixated horizontal 0.2° ± 0.4°, vertical −0.05 ± 0.28°, p < 0.05, for each monkey and dimension tested separately.

### Analysis of BARMs and microsaccades

#### Detection of blinks

We detected blinks offline using homemade programs based on Matlab 2012a (MathWorks, Natick, MA, USA). The first step was to filter eye position data using a Savitzky-Golay filter with polynomial order 1 and a frame size of 15 samples.

We then identified the characteristic blink-associated changes of eye position as decribed by (Goossens and Van Opstal, 2000) focusing on the vertical velocity profile. The velocity profile was considered to reflect a blink-associated eye movement if it had two opposite peaks, a first negative one, and a second positive one, both exceeding 20°/s within a time interval of less than 50 ms as exemplified in (Fig. 1). We additionally considered a criterion based on the acceleration profile since it enhances distinguishing blinks from microsaccades. Blink-associated eye movements had two large acceleration peaks of 56.3 ± 65.4°/s² (mean ± std) in M1 and 101.2 ± 75.2°/s² in M2, whereas microsaccades exhibited comparatively low peaks of 5.7 ± 9.7°/s² in M1 and 6.4 ± 18.8°/s² in M2. Both the velocity and the acceleration criteria had to be met. Once a blink-associated eye movement was detected its onset and offset was determined by a velocity threshold (>2°/s). we induced safety time intervals of around 7 samples (=7 ms) before and after the blinks and microsaccades, similar to our human study, to guarantee that the pre- and post- blink or microsaccade eye positions exclude the moment when the eye is moving. Visual inspection of all blink-associated eye movements and their timing as identified by the automatic procedure ensured that it had not misinterpreted one of the rare artifacts as eye movements.

#### Comparison of BARMs and microsaccades regression slopes and induced variance

Blinks and microsaccades were analyzed separately. The eye movements were pooled from both monkeys and analyzed separately for the horizontal and vertical dimension. We collected around 5,700 blinks and 77,000 microsaccades.

In order to accommodate the large difference in the size of the blink and microsaccade samples we resorted to a bootstrapping procedure to obtain matching numbers of blinks and microsaccades before subjecting them to the regression analysis measurements of variance.

From each pool we randomly drew 5000 samples per iteration, a process that was repeated 100 times, each time replacing the drawn samples. The BARM and microsccade data subsets resulting from each iteration were subjected to a regression of movement amplitude as a function of movement starting position. For each iteration, we determined the regression parameters for the full starting position range and the standard deviation of microsaccade and BARM amplitudes starting from a range of −0.3° to 0.3°. The standard deviation was our measure of the induced variability of the BARMs and microsaccades respectively. We compared the distributions of standard deviations and the regression slopes for microsaccades and BARMs produced by the 100 iterations using a two-samples t-test.

#### Analysis related to the comparison between BARMs and microsaccades resetting.

Figure 2C is a plot of the mean length of movement vectors for the three vector fields shown in Fig. 2A,B as function of the pixelled (0.2° × 0.2°) starting position. The data were fitted by exponential functions separately for BARMs, microsaccades and the subtraction field vectors. The fitting parameters were as follows. BARMS: offset (95% confidence intervals given in parentheses) = 0.112° (0.06042, 0.1635) and steepness = 1.93 (1.48, 2.38), microsaccades: 0.05086° (0.02719, 0.07452) and 2.213 (1.813, 2.613), BARMs-microsaccades: 0.08239° (0.04023, 0.1245) and 1.734 (1.213, 2.254).

Figure 3C contains data pooled from both monkeys and for the eccentric fixation on both monitors. These data correspond to the ones presented in Fig. 3A,B. The fitting parameters for Fig. 3C were as follows. BARMS: offset (95% confidence intervals given in parentheses) = 0.07° (0.02439, 0.1097) and steepness = 2.198° (1.62, 2.775), microsaccades: 0.06803 (0.04572, 0.09035) and 1.483 (1.191, 1.775), BARMs-microsaccades: 0.03841 (0.01111, 0.06571) and 2.288 (1.648, 2.929).

Panels D and E depict the results of a bootstrapping based comparison needed to accommodate the large difference in the size of the blink and microsaccade samples. In order to generate the comparison of starting positions of BARMs and microsaccades in Fig. 2D we proceeded as follows: from each pool, we randomly drew 500 samples per iteration, a process that was repeated 100 times, each time replacing the drawn samples. We took the mean of the BARM and microsaccade subsets resulting from each iteration. This resulted in distributions of 100 means of BARM and microsaccades respectively. We tested if the two distributions were the same or differing using a two samples t-test. The same procedure was applied to the data obtained during fixation on eccentric monitors. The results are depicted in Fig. 3D.

In order to generate the spatial distribution of landing points of BARMs and microsaccades in spatial elements of 0.2 × 0.2° shown in Fig. 2E we proceeded as follows: In order to generate one landing plot, we randomly drew 500 samples per iteration. This process was repeated 100 times, each time replacing the drawn samples. The plots in Fig. 2E are showing landing points of all BARMs and microsaccades. In order to test if the plots for BARMs and microsaccades were different, we ran 100 2D cross-correlation between randomly chosen individual landing plot maps of microsaccades and BARMs. The resulting distribution of 2D shifts was then compared with the distribution of 2D shifts generated by 100 2D cross-correlations between the individual landing point maps of microsaccades, taken as measure of noise. The two distributions obtained were tested resorting to a two samples t-test.
